# *catena*-Poly[bis­[*cis*-dipyrimidine-*trans*-dithio­cyanato­iron(II)]-di-μ-pyrimidine-[*trans*-dithio­cyanato­iron(II)]-di-μ-pyrimidine]

**DOI:** 10.1107/S1600536809005509

**Published:** 2009-02-21

**Authors:** Mario Wriedt, Sina Sellmer, Inke Jess, Christian Näther

**Affiliations:** a Institut für Anorganische Chemie, Christian-Albrechts-Universität Kiel, Max-Eyth-Strasse 2, D-24118 Kiel, Germany

## Abstract

In the crystal structure of the title compound, [Fe_3_(NCS)_6_(C_4_H_4_N_2_)_8_]_*n*_, each iron(II) cation is coordinated by four *N*-bonded pyrimidine ligands and two *N*-bonded thio­cyanate anions in a distorted octa­hedral environment. The asymmetric unit consists of one iron cation located on a crystallographic center of inversion, as well as one iron cation, three thio­cyanate anions and four pyrimidine ligands occupying general positions. The structure consists of square secondary building units (SBUs) with an Fe atom at each corner, which are μ-*N* ^1^:*N* ^3^-bridged by the pyrimidine ligands. The SBUs are linked into infinite chains running in the *c*-axis direction *via* common opposite corners.

## Related literature

For related pyrimidine structures, see: Lloret *et al.* (1998 ▶); Näther *et al.* (2007 ▶); Näther & Jess (2004 ▶). For general background, see: Näther & Greve (2003 ▶); Näther, Wriedt & Jess (2003 ▶); Wriedt *et al.* (2008 ▶, 2009 ▶).
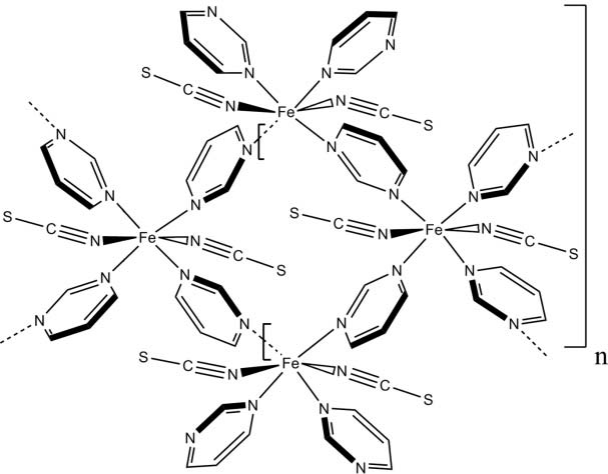


## Experimental

### 

#### Crystal data

[Fe_3_(NCS)_6_(C_4_H_4_N_2_)_8_]
*M* *_r_* = 1156.77
Monoclinic, 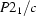

*a* = 18.256 (1) Å
*b* = 16.2855 (9) Å
*c* = 8.2765 (4) Å
β = 100.042 (7)°
*V* = 2423.0 (2) Å^3^
*Z* = 2
Mo *K*α radiation
μ = 1.20 mm^−1^
*T* = 170 K
0.12 × 0.10 × 0.07 mm

#### Data collection

Stoe IPDS-1 diffractometer
Absorption correction: numerical (*X-SHAPE* and *X-RED32*; Stoe, 2008 ▶) *T*_min_ = 0.859, *T*_max_ = 0.912
20463 measured reflections
4615 independent reflections
3795 reflections with *I* > 2σ(*I*)
*R*_int_ = 0.064

#### Refinement

*R*[*F* ^2^ > 2σ(*F* ^2^)] = 0.051
*wR*(*F* ^2^) = 0.124
*S* = 1.13
4615 reflections
314 parameters
H-atom parameters constrained
Δρ_max_ = 0.95 e Å^−3^
Δρ_min_ = −0.41 e Å^−3^

### 

Data collection: *X-AREA* (Stoe, 2008 ▶); cell refinement: *X-AREA*; data reduction: *X-AREA*; program(s) used to solve structure: *SHELXS97* (Sheldrick, 2008 ▶); program(s) used to refine structure: *SHELXL97* (Sheldrick, 2008 ▶); molecular graphics: *XP* in *SHELXTL* (Sheldrick, 2008 ▶); software used to prepare material for publication: *XCIF* in *SHELXTL*.

## Supplementary Material

Crystal structure: contains datablocks I, global. DOI: 10.1107/S1600536809005509/im2102sup1.cif

Structure factors: contains datablocks I. DOI: 10.1107/S1600536809005509/im2102Isup2.hkl

Additional supplementary materials:  crystallographic information; 3D view; checkCIF report

## Figures and Tables

**Table 1:** Selected bond lengths (Å)

Fe1—N41	2.075 (3)
Fe1—N51	2.089 (3)
Fe1—N1	2.252 (3)
Fe1—N31	2.254 (3)
Fe1—N21	2.262 (3)
Fe1—N11	2.304 (3)
Fe2—N61	2.075 (3)
Fe2—N61^i^	2.075 (3)
Fe2—N2	2.252 (3)
Fe2—N2^i^	2.252 (3)
Fe2—N32^ii^	2.291 (3)
Fe2—N32^iii^	2.291 (3)

Symmetry codes: (i) 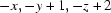; (ii) 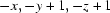; (iii) 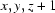.

**Fig. 1.**
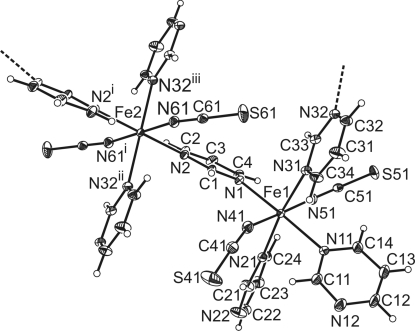
Molecular structure of the repeating unit of the title compound showing the labelling scheme. Displacement ellipsoids drawn at the 50% probability level. Symmetry codes: i = -x, -y + 1, -z + 2; ii = x, y, z + 1; iii = -x, -y + 1, -z + 1.

**Fig. 2.**
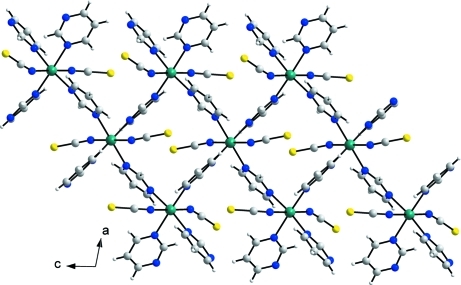
: Crystal structure of the title compound viewed along the b-axis.

## supplementary crystallographic information

### Comment

In our ongoing investigation on the syntheses, structures and properties of new coordination polymers based on paramagnetic metal pseudohalides and N-donor ligands, we have demonstrated that new ligand deficient coordination polymers with interesting magnetic interactions can be conveniently prepared by thermal decomposition of suitable ligand rich precursor compounds (Näther & Greve, 2003 and Wriedt *et al.*,  2009). In further investigations we have reacted iron(II) sulfate heptahydrate and potassium thiocyanate with pyrimidine leading to the formation of single crystals of the title compound.

The 3:8 title compound [(Fe(SCN)_2_)_3_(µ-pyrimidine)_8_(pyrimidine)_2_]_n_ structurally (Fig. 1) represents a chain-like coordination polymer consisting of square SBU's with shared opposite corners as the characteristic motif. Each square SBU contains four iron(II) cations in its corners that are µ-1,3-(*N*,*N'*) bridged by pyrimidine ligands. The commonly used corner iron cations are located on crystallographic centers of inversion and are each octahedrally coordinated by four bridging pyrimidine ligands and two symmetry related terminal *N*-bonded thiocyanate anions. The iron cations in general positions are each octahedrally coordinated by two bridging pyrimidine ligands as well as two terminal *N*-bonded pyrimidine ligands and two terminal *N*-bonded thiocyanate anions (Fig 2). The Fe—NCS distances amount to 2.075 (3) and 2.089 (3) Å and the Fe—N_pyrimidine_ distances range from 2.252 (3) to 2.304 (3) Å. The angles around the iron cations range from 86.9 (1) to 180.0°. The shortest intra- and interchain Fe···Fe distances measure to 6.2508 (6) and 8.7620 (8)Å, respectively.

### Experimental

FeSO_4_.7H_2_O and pyrimidine were obtained from Alfa Aesar, KSCN was obtained from Fluka. 0.25 mmol (69.5 mg) FeSO_4_ × 7 H_2_O, 0.5 mmol (48.6 mg) KSCN and 9 mmol (720.8 mg) pyrimidine were transfered into a test-tube. In a solvent-free reaction at room temperature without stirring red block-shaped single crystals of the title compound were obtained after 2 weeks.

### Refinement

All H atoms were located from difference map but were positioned with idealized geometry and were refined isotropic with *U*_eq_(H) = 1.2 *U*_eq_(C) of the parent atom using a riding model with C—H = 0.95 Å.

### Crystal data

**Table:**

[Fe_3_(NCS)_6_(C_4_H_4_N_2_)_8_]	*F*(000) = 1176
*M**_r_* = 1156.77	*D*_x_ = 1.586 Mg m^−^^3^
Monoclinic, *P*2_1_/*c*	Mo *K*α radiation, λ = 0.71073 Å
Hall symbol: -P 2ybc	Cell parameters from 8000 reflections
*a* = 18.256 (1) Å	θ = 8.3–27.2°
*b* = 16.2855 (9) Å	µ = 1.20 mm^−^^1^
*c* = 8.2765 (4) Å	*T* = 170 K
β = 100.042 (7)°	Block, red
*V* = 2423.0 (2) Å^3^	0.12 × 0.10 × 0.07 mm
*Z* = 2	

### Data collection

**Table:**

Stoe IPDS-1 diffractometer	4615 independent reflections
Radiation source: fine-focus sealed tube	3795 reflections with *I* > 2σ(*I*)
graphite	*R*_int_ = 0.064
φ scans	θ_max_ = 26.0°, θ_min_ = 2.6°
Absorption correction: numerical (*X-SHAPE* and *X-RED32*; Stoe, 2008)	*h* = −22→22
*T*_min_ = 0.859, *T*_max_ = 0.912	*k* = −20→20
20463 measured reflections	*l* = −10→10

### Refinement

**Table:**

Refinement on *F*^2^	Secondary atom site location: difference Fourier map
Least-squares matrix: full	Hydrogen site location: inferred from neighbouring sites
*R*[*F*^2^ > 2σ(*F*^2^)] = 0.051	H-atom parameters constrained
*wR*(*F*^2^) = 0.124	*w* = 1/[σ^2^(*F*_o_^2^) + (0.0419*P*)^2^ + 8.0294*P*] where *P* = (*F*_o_^2^ + 2*F*_c_^2^)/3
*S* = 1.13	(Δ/σ)_max_ < 0.001
4615 reflections	Δρ_max_ = 0.95 e Å^−^^3^
314 parameters	Δρ_min_ = −0.41 e Å^−^^3^
0 restraints	Extinction correction: *SHELXL97* (Sheldrick, 2008), Fc^*^=kFc[1+0.001xFc^2^λ^3^/sin(2θ)]^-1/4^
Primary atom site location: structure-invariant direct methods	Extinction coefficient: 0.0024 (5)

### Special details

**Table:**

Geometry. All e.s.d.'s (except the e.s.d. in the dihedral angle between two l.s. planes) are estimated using the full covariance matrix. The cell e.s.d.'s are taken into account individually in the estimation of e.s.d.'s in distances, angles and torsion angles; correlations between e.s.d.'s in cell parameters are only used when they are defined by crystal symmetry. An approximate (isotropic) treatment of cell e.s.d.'s is used for estimating e.s.d.'s involving l.s. planes.
Refinement. Refinement of *F*^2^ against ALL reflections. The weighted *R*-factor *wR* and goodness of fit *S* are based on *F*^2^, conventional *R*-factors *R* are based on *F*, with *F* set to zero for negative *F*^2^. The threshold expression of *F*^2^ > σ(*F*^2^) is used only for calculating *R*-factors(gt) *etc*. and is not relevant to the choice of reflections for refinement. *R*-factors based on *F*^2^ are statistically about twice as large as those based on *F*, and *R*- factors based on ALL data will be even larger.

### Fractional atomic coordinates and isotropic or equivalent isotropic displacement parameters (Å^2^)

**Table:**

	*x*	*y*	*z*	*U*_iso_*/*U*_eq_	
Fe1	0.26416 (3)	0.48712 (3)	0.61108 (6)	0.01115 (16)	
Fe2	0.0000	0.5000	1.0000	0.01002 (18)	
N41	0.26059 (19)	0.5829 (2)	0.7749 (4)	0.0202 (7)	
C41	0.2859 (2)	0.6276 (2)	0.8790 (5)	0.0182 (8)	
S41	0.32547 (8)	0.68848 (8)	1.02316 (17)	0.0418 (4)	
N51	0.26521 (18)	0.3912 (2)	0.4439 (4)	0.0184 (7)	
C51	0.26229 (19)	0.3726 (2)	0.3063 (5)	0.0127 (7)	
S51	0.25824 (6)	0.35068 (7)	0.11413 (12)	0.0241 (3)	
N61	0.00127 (18)	0.5933 (2)	0.8299 (4)	0.0167 (7)	
C61	0.0140 (2)	0.6208 (2)	0.7072 (5)	0.0146 (8)	
S61	0.03129 (7)	0.65765 (7)	0.53635 (14)	0.0292 (3)	
N1	0.17147 (16)	0.42202 (19)	0.7062 (4)	0.0131 (6)	
N2	0.07898 (17)	0.42630 (19)	0.8767 (4)	0.0131 (6)	
C1	0.12769 (19)	0.4614 (2)	0.7952 (4)	0.0130 (7)	
H1	0.1317	0.5195	0.8008	0.016*	
C2	0.0754 (2)	0.3440 (2)	0.8720 (5)	0.0165 (8)	
H2	0.0432	0.3166	0.9330	0.020*	
C3	0.1170 (2)	0.2978 (2)	0.7813 (5)	0.0179 (8)	
H3	0.1133	0.2397	0.7768	0.021*	
C4	0.1643 (2)	0.3399 (2)	0.6977 (5)	0.0164 (8)	
H4	0.1927	0.3099	0.6320	0.020*	
N11	0.36347 (17)	0.5483 (2)	0.5208 (4)	0.0157 (7)	
N12	0.4695 (2)	0.6360 (2)	0.5800 (5)	0.0264 (8)	
C11	0.4082 (2)	0.6011 (3)	0.6157 (5)	0.0212 (9)	
H11	0.3949	0.6149	0.7183	0.025*	
C12	0.4872 (2)	0.6144 (3)	0.4348 (6)	0.0275 (10)	
H12	0.5309	0.6369	0.4048	0.033*	
C13	0.4452 (3)	0.5618 (3)	0.3286 (6)	0.0289 (10)	
H13	0.4584	0.5482	0.2258	0.035*	
C14	0.3827 (2)	0.5293 (3)	0.3769 (5)	0.0231 (9)	
H14	0.3525	0.4923	0.3056	0.028*	
N21	0.34829 (17)	0.4171 (2)	0.7928 (4)	0.0150 (7)	
N22	0.4465 (2)	0.4159 (2)	1.0257 (5)	0.0285 (9)	
C21	0.3925 (2)	0.4525 (3)	0.9200 (5)	0.0216 (9)	
H21	0.3847	0.5093	0.9370	0.026*	
C22	0.4557 (2)	0.3357 (3)	1.0026 (5)	0.0249 (9)	
H22	0.4936	0.3074	1.0746	0.030*	
C23	0.4125 (2)	0.2927 (3)	0.8790 (5)	0.0219 (9)	
H23	0.4191	0.2354	0.8658	0.026*	
C24	0.3587 (2)	0.3362 (2)	0.7736 (5)	0.0150 (8)	
H24	0.3284	0.3080	0.6859	0.018*	
N31	0.19048 (17)	0.5567 (2)	0.4101 (4)	0.0155 (7)	
N32	0.09789 (17)	0.56059 (19)	0.1687 (4)	0.0143 (7)	
C31	0.1369 (2)	0.5230 (2)	0.2998 (5)	0.0151 (8)	
H31	0.1253	0.4670	0.3159	0.018*	
C32	0.1156 (2)	0.6395 (2)	0.1491 (5)	0.0184 (8)	
H32	0.0906	0.6684	0.0558	0.022*	
C33	0.1689 (2)	0.6805 (3)	0.2591 (6)	0.0244 (9)	
H33	0.1799	0.7368	0.2448	0.029*	
C34	0.2051 (2)	0.6362 (2)	0.3899 (5)	0.0203 (9)	
H34	0.2417	0.6627	0.4684	0.024*	

### Atomic displacement parameters (Å^2^)

**Table:**

	*U*^11^	*U*^22^	*U*^33^	*U*^12^	*U*^13^	*U*^23^
Fe1	0.0097 (3)	0.0137 (3)	0.0102 (3)	−0.00061 (19)	0.0022 (2)	−0.0013 (2)
Fe2	0.0084 (3)	0.0124 (4)	0.0098 (4)	0.0004 (3)	0.0029 (3)	0.0014 (3)
N41	0.0200 (17)	0.0208 (18)	0.0212 (18)	−0.0004 (14)	0.0073 (15)	−0.0038 (14)
C41	0.0174 (18)	0.0167 (19)	0.020 (2)	0.0057 (15)	0.0032 (16)	−0.0026 (16)
S41	0.0447 (7)	0.0309 (7)	0.0414 (7)	0.0109 (5)	−0.0158 (6)	−0.0206 (6)
N51	0.0190 (17)	0.0198 (17)	0.0169 (17)	0.0001 (13)	0.0047 (14)	−0.0014 (14)
C51	0.0103 (16)	0.0106 (17)	0.0170 (19)	0.0005 (13)	0.0015 (14)	−0.0018 (14)
S51	0.0343 (6)	0.0272 (5)	0.0112 (5)	−0.0026 (4)	0.0052 (4)	−0.0027 (4)
N61	0.0162 (16)	0.0181 (17)	0.0161 (17)	0.0015 (12)	0.0035 (13)	0.0033 (13)
C61	0.0127 (17)	0.0131 (17)	0.0178 (19)	0.0037 (14)	0.0022 (15)	−0.0002 (15)
S61	0.0441 (7)	0.0281 (6)	0.0189 (5)	0.0067 (5)	0.0147 (5)	0.0085 (4)
N1	0.0085 (14)	0.0158 (16)	0.0155 (15)	−0.0002 (11)	0.0034 (12)	−0.0004 (12)
N2	0.0114 (14)	0.0153 (15)	0.0132 (15)	0.0004 (12)	0.0039 (12)	−0.0001 (12)
C1	0.0110 (16)	0.0159 (18)	0.0126 (17)	0.0012 (14)	0.0035 (14)	0.0007 (14)
C2	0.0151 (18)	0.0142 (18)	0.022 (2)	−0.0018 (14)	0.0075 (16)	0.0016 (15)
C3	0.0166 (18)	0.0114 (17)	0.026 (2)	−0.0026 (14)	0.0042 (16)	−0.0024 (15)
C4	0.0124 (17)	0.0175 (19)	0.020 (2)	0.0009 (14)	0.0039 (15)	−0.0038 (15)
N11	0.0107 (14)	0.0210 (17)	0.0153 (16)	−0.0021 (12)	0.0015 (13)	0.0017 (13)
N12	0.0231 (18)	0.032 (2)	0.027 (2)	−0.0136 (15)	0.0106 (16)	−0.0069 (16)
C11	0.0184 (19)	0.026 (2)	0.021 (2)	−0.0072 (16)	0.0072 (17)	−0.0056 (16)
C12	0.024 (2)	0.038 (3)	0.024 (2)	−0.0139 (19)	0.0140 (18)	−0.0027 (19)
C13	0.028 (2)	0.040 (3)	0.021 (2)	−0.012 (2)	0.0124 (19)	−0.0024 (19)
C14	0.024 (2)	0.031 (2)	0.015 (2)	−0.0097 (17)	0.0052 (17)	−0.0062 (17)
N21	0.0131 (15)	0.0181 (16)	0.0139 (15)	−0.0007 (12)	0.0026 (13)	0.0022 (13)
N22	0.0267 (19)	0.026 (2)	0.0274 (19)	−0.0014 (15)	−0.0108 (16)	0.0036 (16)
C21	0.025 (2)	0.021 (2)	0.0167 (19)	0.0001 (16)	−0.0026 (17)	0.0009 (16)
C22	0.021 (2)	0.027 (2)	0.026 (2)	0.0033 (17)	−0.0006 (18)	0.0084 (18)
C23	0.022 (2)	0.022 (2)	0.021 (2)	0.0063 (16)	0.0013 (17)	0.0055 (16)
C24	0.0157 (18)	0.0183 (19)	0.0114 (18)	0.0018 (14)	0.0036 (15)	0.0022 (14)
N31	0.0115 (15)	0.0181 (16)	0.0163 (16)	−0.0014 (12)	0.0011 (13)	0.0007 (13)
N32	0.0116 (15)	0.0154 (16)	0.0156 (16)	−0.0005 (12)	0.0012 (13)	−0.0015 (12)
C31	0.0112 (17)	0.0169 (18)	0.0165 (18)	0.0004 (14)	0.0010 (15)	−0.0004 (15)
C32	0.0155 (18)	0.0185 (19)	0.020 (2)	0.0003 (15)	−0.0008 (16)	0.0061 (15)
C33	0.023 (2)	0.017 (2)	0.031 (2)	−0.0046 (16)	−0.0027 (18)	0.0032 (17)
C34	0.0185 (19)	0.020 (2)	0.020 (2)	−0.0045 (15)	−0.0032 (16)	−0.0030 (16)

### Geometric parameters (Å, °)

**Table:**

Fe1—N41	2.075 (3)	N12—C11	1.334 (5)
Fe1—N51	2.089 (3)	N12—C12	1.344 (6)
Fe1—N1	2.252 (3)	C11—H11	0.9500
Fe1—N31	2.254 (3)	C12—C13	1.364 (6)
Fe1—N21	2.262 (3)	C12—H12	0.9500
Fe1—N11	2.304 (3)	C13—C14	1.378 (6)
Fe2—N61	2.075 (3)	C13—H13	0.9500
Fe2—N61^i^	2.075 (3)	C14—H14	0.9500
Fe2—N2	2.252 (3)	N21—C21	1.340 (5)
Fe2—N2^i^	2.252 (3)	N21—C24	1.345 (5)
Fe2—N32^ii^	2.291 (3)	N22—C22	1.334 (6)
Fe2—N32^iii^	2.291 (3)	N22—C21	1.339 (5)
N41—C41	1.161 (5)	C21—H21	0.9500
C41—S41	1.621 (4)	C22—C23	1.371 (6)
N51—C51	1.171 (5)	C22—H22	0.9500
C51—S51	1.619 (4)	C23—C24	1.388 (5)
N61—C61	1.169 (5)	C23—H23	0.9500
C61—S61	1.618 (4)	C24—H24	0.9500
N1—C1	1.341 (5)	N31—C31	1.335 (5)
N1—C4	1.344 (5)	N31—C34	1.338 (5)
N2—C1	1.335 (5)	N32—C31	1.338 (5)
N2—C2	1.343 (5)	N32—C32	1.343 (5)
C1—H1	0.9500	N32—Fe2^iv^	2.291 (3)
C2—C3	1.380 (6)	C31—H31	0.9500
C2—H2	0.9500	C32—C33	1.382 (6)
C3—C4	1.379 (6)	C32—H32	0.9500
C3—H3	0.9500	C33—C34	1.372 (6)
C4—H4	0.9500	C33—H33	0.9500
N11—C14	1.336 (5)	C34—H34	0.9500
N11—C11	1.342 (5)		
			
N41—Fe1—N51	178.69 (13)	N1—C4—H4	118.9
N41—Fe1—N1	90.82 (13)	C3—C4—H4	118.9
N51—Fe1—N1	88.50 (12)	C14—N11—C11	116.2 (3)
N41—Fe1—N31	91.24 (13)	C14—N11—Fe1	122.3 (3)
N51—Fe1—N31	87.72 (12)	C11—N11—Fe1	121.4 (3)
N1—Fe1—N31	96.10 (11)	C11—N12—C12	115.4 (4)
N41—Fe1—N21	92.26 (13)	N12—C11—N11	126.3 (4)
N51—Fe1—N21	88.85 (12)	N12—C11—H11	116.8
N1—Fe1—N21	89.70 (11)	N11—C11—H11	116.8
N31—Fe1—N21	173.18 (12)	N12—C12—C13	123.0 (4)
N41—Fe1—N11	90.18 (13)	N12—C12—H12	118.5
N51—Fe1—N11	90.56 (13)	C13—C12—H12	118.5
N1—Fe1—N11	176.83 (11)	C12—C13—C14	117.1 (4)
N31—Fe1—N11	86.88 (11)	C12—C13—H13	121.5
N21—Fe1—N11	87.25 (11)	C14—C13—H13	121.5
N61—Fe2—N61^i^	180.000 (1)	N11—C14—C13	122.0 (4)
N61—Fe2—N2	89.96 (12)	N11—C14—H14	119.0
N61^i^—Fe2—N2	90.04 (12)	C13—C14—H14	119.0
N61—Fe2—N2^i^	90.04 (12)	C21—N21—C24	115.9 (3)
N61^i^—Fe2—N2^i^	89.96 (12)	C21—N21—Fe1	123.5 (3)
N2—Fe2—N2^i^	180.000 (1)	C24—N21—Fe1	120.6 (2)
N61—Fe2—N32^ii^	90.10 (12)	C22—N22—C21	116.0 (4)
N61^i^—Fe2—N32^ii^	89.90 (12)	N22—C21—N21	126.6 (4)
N2—Fe2—N32^ii^	89.27 (11)	N22—C21—H21	116.7
N2^i^—Fe2—N32^ii^	90.73 (11)	N21—C21—H21	116.7
N61—Fe2—N32^iii^	89.90 (12)	N22—C22—C23	122.4 (4)
N61^i^—Fe2—N32^iii^	90.10 (12)	N22—C22—H22	118.8
N2—Fe2—N32^iii^	90.73 (11)	C23—C22—H22	118.8
N2^i^—Fe2—N32^iii^	89.27 (11)	C22—C23—C24	117.4 (4)
N32^ii^—Fe2—N32^iii^	180.000 (1)	C22—C23—H23	121.3
C41—N41—Fe1	154.5 (3)	C24—C23—H23	121.3
N41—C41—S41	177.0 (4)	N21—C24—C23	121.6 (4)
C51—N51—Fe1	146.5 (3)	N21—C24—H24	119.2
N51—C51—S51	177.7 (3)	C23—C24—H24	119.2
C61—N61—Fe2	153.7 (3)	C31—N31—C34	116.8 (3)
N61—C61—S61	179.3 (4)	C31—N31—Fe1	124.8 (3)
C1—N1—C4	116.2 (3)	C34—N31—Fe1	118.2 (2)
C1—N1—Fe1	121.5 (2)	C31—N32—C32	115.6 (3)
C4—N1—Fe1	121.5 (3)	C31—N32—Fe2^iv^	123.0 (3)
C1—N2—C2	116.5 (3)	C32—N32—Fe2^iv^	121.2 (2)
C1—N2—Fe2	122.4 (2)	N31—C31—N32	126.1 (4)
C2—N2—Fe2	120.9 (3)	N31—C31—H31	117.0
N2—C1—N1	125.9 (3)	N32—C31—H31	117.0
N2—C1—H1	117.1	N32—C32—C33	122.7 (4)
N1—C1—H1	117.1	N32—C32—H32	118.7
N2—C2—C3	122.1 (4)	C33—C32—H32	118.7
N2—C2—H2	119.0	C34—C33—C32	116.9 (4)
C3—C2—H2	119.0	C34—C33—H33	121.6
C4—C3—C2	117.0 (4)	C32—C33—H33	121.6
C4—C3—H3	121.5	N31—C34—C33	121.9 (4)
C2—C3—H3	121.5	N31—C34—H34	119.0
N1—C4—C3	122.2 (4)	C33—C34—H34	119.0

Symmetry codes: (i) −*x*, −*y*+1, −*z*+2; (ii) −*x*, −*y*+1, −*z*+1; (iii) *x*, *y*, *z*+1; (iv) *x*, *y*, *z*−1.
